# Enhancing Stability and Antioxidant Activity of Resveratrol-Loaded Emulsions by Ovalbumin–Dextran Conjugates

**DOI:** 10.3390/foods13081246

**Published:** 2024-04-19

**Authors:** Wen Zhang, Lingli Meng, Xinyi Lv, Limin Wang, Pei Zhao, Jinrong Wang, Xinping Zhang, Jinyu Chen, Zijian Wu

**Affiliations:** Tianjin Key Laboratory of Food Science and Biotechnology, School of Biotechnology and Food Science, Tianjin University of Commerce, Tianjin 300134, Chinawangjr@tjcu.edu.cn (J.W.);

**Keywords:** antioxidant, emulsion, ovalbumin–dextran conjugate, resveratrol, stability

## Abstract

A reliable strategy for improving the stability and shelf life of protein-stabilized systems is by covalently attaching the protein onto a polysaccharide. In this study, ovalbumin (OVA) was modified with dextran (DEX) of different molecular weights by the Maillard reaction, and was used to enhance the stability of emulsions loaded with resveratrol. The surface hydrophobicity, thermal stability, and FT-IR spectroscopy of the OVA–DEX conjugates were evaluated. The results showed that the surface hydrophobicity of OVA decreased, while the thermal stability of OVA was significantly improved after DEX covalent modification. The OVA–DEX1k-stabilized emulsion exhibited high encapsulation efficiency of resveratrol, with the value of 89.0%. In addition, OVA–DEX was considerably more effective in droplet stabilization against different environmental stresses (heat, pH, and ionic strength). After 28 days of storage at 25 °C, the OVA-stabilized emulsion showed faster decomposition of resveratrol, whereas the OVA–DEX-conjugate-stabilized emulsion had approximately 73% retention of resveratrol. Moreover, the antioxidant activity of resveratrol-loaded emulsions stabilized by OVA–DEX was higher during storage under different temperatures. These results proved that the OVA–DEX conjugates had the potential to form stable, food-grade emulsion-based delivery systems against environmental stresses, which strongly supports their potential in the field of food and biomedical applications.

## 1. Introduction

Resveratrol, a hydrophobic non-flavonoid polyphenolic compound, is widely present in berries, nuts, and tubers [1]. Resveratrol has gained significant attention in recent years due to its potential health benefits, including antioxidant, anti-inflammatory, anti-tumor, cardioprotective, and neuroprotective activities [2,3]. Despite its numerous advantages, resveratrol faces certain limitations that hinder its applicability in the food and pharmaceutical industries [4]. One major obstacle is its poor solubility in water, which makes it difficult to dissolve in aqueous formulations. Additionally, resveratrol has low stability and can readily degrade when exposed to acidic conditions or thermal treatment, diminishing its effectiveness over time [4]. Hence, a suitable vehicle should be performed to enhance the solubility and stability of resveratrol for the purpose of ultimately maximizing its beneficial effects.

Emulsion is generally known for their ability to enhance the water solubility, stability, and bioavailability of liposoluble bioactive compounds [5,6]. In emulsions, proteins are commonly employed as emulsifiers [7]. The hydrophobic regions of protein molecules can adsorb onto the surface of hydrophobic substances, aiding in their dispersion in water. However, proteins are susceptible to thermal treatment, changes in pH and ionic strength, and may even undergo denaturation and aggregation, leading to phase separation in protein-stabilized systems [6,7,8]. To improve the stability of emulsions, protein–polysaccharide conjugation is utilized. The protein component of protein–polysaccharides conjugates adsorbs onto the surface of oil droplets, stabilizing the oil–water interface and preventing the further coalescence of the oil droplets. On the other hand, the polysaccharide component of the copolymer not only possesses water solubility but also creates a strong steric hindrance effect to prevent oil droplet aggregation [9,10]. Thus, protein–polysaccharide conjugates exhibit enhanced stability against heat and changes in pH and ionic strength, and they are commonly used for encapsulating hydrophobic active substances such as curcumin [11], lutein [10], and β-carotene [12]. For example, Xu et al. successfully stabilized β-carotene using a whey protein isolate–beet pectin conjugate, which not only improved the physical stability of the emulsion but also delayed the degradation of β-carotene [12].

Ovalbumin (OVA), a glycosylated globulin derived from chicken egg white [13], possesses excellent functional properties such as emulsification, thickening, gelling, and foaming [14]. It serves as a suitable carrier for bioactive substances, significantly enhancing water solubility and stability of curcumin under light condition [15]. However, OVA has poor thermal stability and may precipitate near its isoelectric point [16]. Glycosylation modification of OVA through the Maillard reaction could effectively improve its processing characteristics, including thermal and pH stability [14,17]. Dextran (DEX) is a reducible polysaccharide that can covalently bind to proteins, enhancing their functional properties such as emulsification and gelling [18,19]. Dextran presents with a variety of molecular weights ranging from 1 to 440 MDa [20], among which dextrans with 1 kDa, 10 kDa, and 70 kDa molecular weights are widely used in the food and pharmaceutical industry due to their satisfactory safety [21], benefits in nutraceuticals encapsulation [20,22,23], and potential effects for health [24,25]. For example, a protamine and BSA–dextran complex emulsion was prepared to improve the oral bioavailability and anti-tumor efficacy of paclitaxel [22]. Fan et al. also demonstrated that β-carotene-loaded nano-emulsions, stabilized with whey protein–dextran conjugates, led to the enhanced in vitro bioaccessibility of β-carotene in the gastrointestinal tract [26]. Although these results suggested that protein–dextran conjugates were suitable for encapsulating liposoluble bioactive compounds, the stability of emulsions against external environmental stresses (such as high temperature, freeze–thawing, different pH condition, and high ionic strength) still need further investigation for their applications in the food processing and handling industry.

The focus of the current work was the utilization of protein–polysaccharide Maillard conjugates (OVA–DEX conjugates) as emulsifiers to modulate the physicochemical stability, especially under external environmental stresses, and antioxidant capacity of resveratrol-loaded emulsions in vitro. In this study, the structure of OVA–DEX conjugates was characterized by sodium dodecylsulfate–polyacrylamide gel electrophoresis (SDS-PAGE) and Fourier-transform infrared (FT-IR) spectroscopy. The effect of OVA–DEX on the stability and antioxidant activity of resveratrol-loaded emulsion was further investigated by particle size, droplet size, polydispersity index (PDI), turbidity value, and resveratrol residual content. This study presented detailed information that using OVA–DEX conjugates as emulsifiers led to notable improvements in the resistance of emulsions to specific environmental factors, making them ideal for applications in the pharmaceutical and food industries.

## 2. Materials and Methods

### 2.1. Materials

Ovalbumin (OVA, ≥90%), dextran (DEX, ≥95%, 1–70 kDa), resveratrol (≥98%), medium chain triglyceride (MCT), and potassium ferricyanide were purchased from Shanghai Yuan Ye Biotech Co., Ltd. (Shanghai, China). ABTS (2,2′-azinobis (3-ethylbenzothiazoline-6-sulfonic acid) ammonium salt) and potassium persulfate were obtained from Aladdin Reagent Co., Ltd. (Shanghai, China). O-Phthalaldehyde (OPA) was purchased from Macklin (Shanghai, China). All other reagents used were of analytical grade.

### 2.2. Preparation and Characterization of OVA–DEX Conjugate

#### 2.2.1. Preparation of OVA–DEX Conjugate

The OVA–DEX conjugate was prepared according to the published method with slight modification [27,28]. Briefly, OVA was dissolved in ultrapure water and stirred magnetically for 3 h at room temperature. It was stored overnight at 4 °C and then centrifuged at 6000 rpm for 10 min at 4 °C to remove any insoluble material. Quantitation of OVA in the solution was determined using UV absorbance at 280 nm (NanoPhotometer-N50, IMPLEN, Germany) [29]. The concentration of OVA in the supernatant was adjusted to 10 mg/mL. DEX (the molecular weight is 1, 10, and 70 kDa, respectively) and OVA were dissolved in ultrapure water at a ratio of 1:1 (*w*/*w*) and stirred at room temperature for 3 h to achieve a concentration of 10 mg/mL of OVA. According to the method reported previously [26,30,31,32], the mixture was freeze-dried and incubated at 60 °C and 79% relative humidity (in a desiccator with a saturated KBr solution) for 24 h, 48 h, and 72 h, respectively. The incubated samples were dialyzed against deionized water at 4 °C [33,34], and then freeze-dried to obtain OVA–DEX conjugates (the conjugates were given the names OVA–DEX1k, OVA–DEX10k, and OVA–DEX70k).

#### 2.2.2. SDS-PAGE

SDS-PAGE was used to confirm the formation of OVA–DEX conjugates based on differences in their molecular weight according to the method reported previously [26]. Briefly, sample solutions with the protein concentration of 2 mg/mL were mixed with 4×SDS loading buffer and heated in boiling water for 10 min. The samples were cooled to room temperature and loaded onto SDS gels previously prepared on a SE-260 system (Hoefer, San Francisco, CA, USA). A voltage of 80 V was applied to the gels for 20 min and then increased to 120 V until the end. The gels were stained with Coomassie brilliant blue R250 for 1 h and scanned for photos after destaining.

#### 2.2.3. Grafting Degree (GD)

The GD was estimated by measuring the free amino groups using OPA assay, according to a published method with slight modification [26]. In brief, 80 mg OPA was dissolved in 2 mL of methanol and mixed with 50 mL of borate buffer (100 mM). Subsequently, 5 mL of SDS (20%, *w*/*v*) and 200 μL of 2-mercaptoethanol were added into the above solution and then diluted to 100 mL with distilled water. Then, 200 μL of the sample (5 mg/mL protein) was mixed with 4 mL OPA reagents and kept at room temperature for 2 min. The absorbance at 340 nm was measured with the Infinite M200 Pro microplate reader (Tecan, Switzerland). The GD was calculated using Equation (1):

(1) $$\mathrm{GD}\;(\%)=\frac{{A_{0}-A}_{1}}{A_{0}}\times 100$$

where A_0_ is the absorbance of OVA and A_1_ is the absorbance of the OVA–DEX conjugates.

#### 2.2.4. Fourier Transforms Infrared (FT-IR) Spectroscopy

The FT-IR spectra of OVA and OVA–DEX conjugates were carried out using an infrared spectrometer equipped with a DTGS detector and single reflection diamond ATR accessory (Bruker, Germany) [35]. For each spectrum, the samples were recorded at a resolution of 4 cm^−1^ from 600 to 4000 cm^−1^.

#### 2.2.5. Surface Hydrophobicity (H_0_)

The H_0_ of OVA and OVA–DEX conjugates was measured with 1-aniline naphthalene-8-sulfonate (ANS) as a fluorescence probe [36]. Briefly, the samples were diluted with phosphate-buffered solution (10.0 mM, pH 7.4) to a concentration of 0.01–0.05 mg/mL. Subsequently, 20 μL of ANS (8.0 mM) was added to 4 mL of the sample solution and reacted at room temperature for 2 min. The fluorescence intensity was recorded by an FL-970 fluorescence spectrophotometer (Techcomp, Shanghai, China) at the excitation wavelength of 370 nm, emission wavelength range of 400–600 nm, and a scanning speed of 600 nm/min. H_0_ was expressed as the slope of the linear regression of the fluorescence intensity against protein concentration.

#### 2.2.6. Heat Stability and Thermal Analysis

The thermal transition temperature was determined using the TG-DTA8122 thermogravimetric analyzer (Rigaku, Tokyo, Japan), following a method previously reported [37]. To elucidate, the samples were placed into an aluminum pan and heated gradually from 30 to 210 °C at a speed of 5 °C/min. A sealed empty aluminum pan was employed as the reference.

The heat stability analysis was conducted according to the published method with slight modification [34,38]. In brief, the powders of OVA and OVA–DEX conjugate were diluted with deionized water to the concentration of 2 mg/mL, respectively. The solutions were separately heated in a water bath at 50, 60, 70, 80, 90, and 100 °C for 10 min, and then cooled down to room temperature. Subsequently, aggregates were precipitated by centrifuging at a speed of 10,000 rpm for 10 min. The absorbance at 280 nm was measured to estimate the concentration of soluble protein in the supernatant. The heat stability of each sample was calculated using Equation (2):

(2) $$\mathrm{Heat}\;\mathrm{stability}\;(\%)=\frac{C_{1}}{C_{0}}\times 100$$

where C_0_ is the initial concentration of OVA and C_1_ indicates the concentration of OVA in the supernatant.

### 2.3. Fabrication and Characterization of Emulsion

#### 2.3.1. Emulsion Fabrication

The resveratrol-loaded emulsions were prepared following the method presented by Wang et al. [11]. In brief, resveratrol was added into a mixed-oil solvent of 90% MCT and 10% ethanol (*v*/*v*) with resveratrol concentration of 5 mg/mL, and the oil solution was stirred in the dark for 1 h until the resveratrol was completely dissolved. Subsequently, the oil solution was added into the conjugate aqueous solution (10 mg/mL protein concentration) with 5% oil volume fraction. The mixture was pre-emulsified by stirring at 10,000 rpm/min for 2 min, and then emulsified using the AH100B high-pressure homogenizer (ATS Engineering Inc., Brampton, ON, Canada) at 600 bar for 7 cycles at 4 °C to obtain fine emulsions.

#### 2.3.2. Encapsulation Efficiency (EE)

For the investigation of the EE, the emulsion was mixed with 75% ethanol in a 19:1 (*v*/*v*) ratio to fully dissolve the resveratrol. The absorbance at 306 nm was measured, and a standard curve was created using the same measurement procedure. The EE was calculated by Equation (3):

(3) $$\mathrm{EE}\;(\%)=\frac{C_{3}}{C_{2}}\times 100$$

where C_3_ is the amount of encapsulated resveratrol and C_2_ is the initial amount of resveratrol.

#### 2.3.3. Droplet Size and Polydispersity Index (PDI)

The freshly prepared emulsions were diluted with deionized water by 400 times to avoid multiple scattering effects before measurement. The droplet size and PDI of emulsions were measured on were assessed by nanoparticle tracking analysis using a Nanosight NS300 system (Malvern, UK) at 25 °C. Three replicate samples per emulsion were evaluated, and each sample was analyzed five times with the instrument to determine mean particle size.

#### 2.3.4. Turbidity

The turbidity was determined at 600 nm using a spectrophotometer according to the method described by Zhu et al. [39]. The emulsions were diluted with the ultrapure water and the results were expressed as Equation (4):

(4) $$T=1.302\times \frac{\mathrm{AV}}{I}$$

where A is the absorbance of the diluted emulsions at 600 nm, V is the dilution factor, and I is the optical path (0.01 m).

#### 2.3.5. Rheological Properties

The rheological properties of the samples were determined following the method presented by Mirarab et al. [40,41,42]. The rheological properties of the emulsions were determined using a MCR 301 rheometer (Anton Paar, Graz, Austria). The measuring system was equipped with a 50 mm diameter parallel plate geometry and the gap between plates was 1 mm. A measure of 1 mL of the emulsion was placed onto the test disk, and a thin layer of silicone oil was placed on the periphery surface of the solution held between the plates to prevent the moisture loss of the samples during the measurement. To determine the linear viscoelastic region (LVR), the strain sweep test was elevated over a strain range of 0.01–100% at a fixed frequency of 1 Hz. Dynamic oscillatory measurements of storage modulus (G′) and loss modulus (G″) were performed from 0.1 to 10 Hz at a fixed strain of 1% within the LVR.

### 2.4. Storage Stability

The freshly prepared emulsions were placed in 50 mL tubes and stored at 25 °C for up to 28 days. For the indicated time points, the emulsion was mixed with 75% ethanol in a 19:1 (*v*/*v*) ratio to fully dissolve the resveratrol. The absorbance at 306 nm was measured, and a standard curve was created using the same measurement procedure. The chemical stability of resveratrol in each sample was expressed as the resveratrol retention and calculated using Equation (5), as follows:

(5) $$\mathrm{Resveratrol}\;\mathrm{retention}\;(\%)=\frac{C_{4}}{C_{3}}\times 100$$

where C_3_ is the amount of resveratrol in freshly prepared samples and C_4_ is the amount of resveratrol in samples after storage for the indicated time points.

### 2.5. Physicochemical Stability

#### 2.5.1. Effect of Thermal Treatment

The freshly prepared resveratrol emulsions were subjected to a water bath at 50 °C or 90 °C for 10 min and then cooled down immediately to room temperature. For the freeze–thaw treatment, the resveratrol emulsions were placed in bottles, frozen, and stored at −18 °C for 2 days. Then, the emulsions were thawed at 25 °C for 12 h. The treated emulsions were stored for particle sizes and PDI measurements.

#### 2.5.2. Effect of pH

The newly produced resveratrol emulsions were diluted to the optimal concentration using the PBS buffer solution (137 mM NaCl, 2.7 mM KCl, 10 mM sodium phosphate dibasic, and 1.8 mM potassium phosphate monobasic, pH 7.4). Then, the pH of the emulsions was adjusted to 3.0, 4.0, 5.0, 6.0, 7.0, 8.0, and 9.0 using 0.1 mol/L HCl or 0.1 mol/L NaOH solutions, separately. Subsequently, the emulsions were transferred to the test tubes and stored at ambient temperature for 5 h for droplet size, PDI, and turbidity value assays.

#### 2.5.3. Effect of Ionic Strength

The resveratrol emulsions were freshly prepared and transferred to glass test tubes. Then, NaCl solutions with concentrations ranging from 1 to 5 mol/L were added to each test tube; subsequently, the above samples were stored at room temperature for droplet size, PDI, and turbidity value measurements.

### 2.6. Antioxidant Activity Test

#### 2.6.1. ABTS Radical Scavenging

The antioxidant activity of emulsion was determined using the ABTS radical scavenging assay as described by Kevij et al., with slight modifications [41]. Briefly, a 7.0 mM ABTS cation radical solution was dissolved in water and allowed to react with a 2.45 mM potassium persulfate solution in a dark room for 16 h. Afterward, the ABTS solution was diluted with water to reach an initial absorbance of 0.7 ± 0.02 at 734 nm. Then, 0.2 mL of the supernatant of each sample was then mixed with 3.0 mL of ABTS working solution. The absorbance was measured at 734 nm, and the scavenging activity was calculated using Equation (6):

(6) $$\mathrm{ABTS}\;\mathrm{scavenging}\;\mathrm{activity}\;(\%)=\frac{{A_{2}-(A_{4}-A}_{3})}{A_{2}}\times 100\;$$

where A_2_ is the absorbance of ABTS solution diluted with water, A_3_ is the absorbance of samples, and A_4_ is the absorbance of the mixture of sample and ABTS solution.

#### 2.6.2. Reducing Power Assay

The ability of emulsions to reduce Fe^3+^ was evaluated following the method of Kevij et al. [41]. Briefly, 0.5 mL of the emulsion sample was mixed with 1.25 mL phosphate buffer (0.2 M, pH 6.6) and 1.25 mL of potassium ferricyanide (10 g/L). After incubation in a water bath for 20 min at 50 °C, 1.25 mL of trichloroacetic acid 10% (*w*/*v*) was added to the mixture. Immediately after shaking, the mixture was centrifuged at 1500× *g* for 10 min and 1.25 mL of the upper layer of each sample was mixed with 1.25 mL of distilled water and 0.25 mL of 0.1% (*w*/*v*) FeCl_3_. After 10 min of incubation at room temperature, the absorbance of the sample solutions was measured at 700 nm. High absorbance indicated high reducing power.

#### 2.6.3. DPPH Scavenging Capacity

The DPPH scavenging capacity of emulsions was measured according to the method reported in [43], with slight modifications. Briefly, 1 mL of emulsion was added to 2 mL of DPPH solution and immediately stirred. After being stored at room temperature for 30 min in the dark, the absorbance value at 517 nm was measured using a spectrophotometer. The DPPH radical scavenging activity (%) was calculated using Equation (7):

(7) $$\mathrm{ABTS}\;\mathrm{scavenging}\;\mathrm{activity}\;(\%)=\frac{{A_{5}-(A_{7}-A}_{6})}{A_{5}}\times 100$$

where A_5_ is the absorbance of DPPH solution diluted with water, A_6_ is the absorbance of samples, and A_7_ is the absorbance of the mixture of sample and DPPH solution.

### 2.7. Statistical Analysis

All samples were conducted at least three times, and the results are given as mean ± standard deviations (SD). Statistical comparisons were determined using the SPSS 20.0 package (IBM, Armonk, NY, USA). Significant between-group differences were determined using one-way analysis of variance and Tukey’s post hoc test. A *p* value < 0.05 was considered to be statistically significant.

## 3. Results and Discussion

### 3.1. Preparation and Characterization of OVA–DEX Conjugate

#### 3.1.1. SDS-PAGE and Grafting Degree Analysis

**Figure 1:**
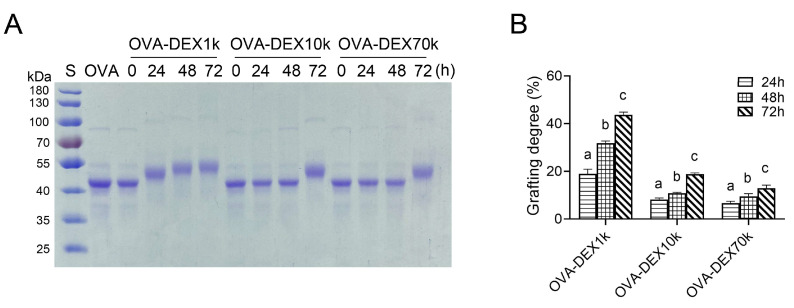
(**A**) SDS-PAGE patterns of native OVA and the OVA–DEX conjugates. (**B**) Grafting degree of the OVA–DEX conjugates after different reaction times. Different letters have significant differences (*p* < 0.05).

The SDS-PAGE electrophoresis analysis was crucial in determining the occurrence of covalent binding and evaluating the molecular weight of the conjugates after grafting [44]. After the grafting reaction of DEX and OVA, SDS-PAGE electrophoresis was performed on the conjugates. The results were shown in Figure 1A: lane 1 served as the standard protein marker and lane 2 represented the OVA protein. It was observed that the OVA–DEX conjugates presented a higher molecular weight than that of OVA, and the molecular weight of the copolymer increased with prolonged reaction time, especially in the OVA–DEX1k and OVA–DEX10k conjugates. The grafting degree analysis further confirmed that DEX with a molecular weight of 1 kDa presented the highest degree of grafting with OVA at the same reaction time, indicating a greater extent of DEX undergoing covalent binding with OVA (Figure 1B). Consistently with the studies conducted by Kim et al. [45] and Yan et al. [35], the results of SDS-PAGE and grafting degree analysis confirmed that there was indeed covalent binding between OVA and DEX through the grafting reaction.

#### 3.1.2. FT-IR Analysis

**Figure 2:**
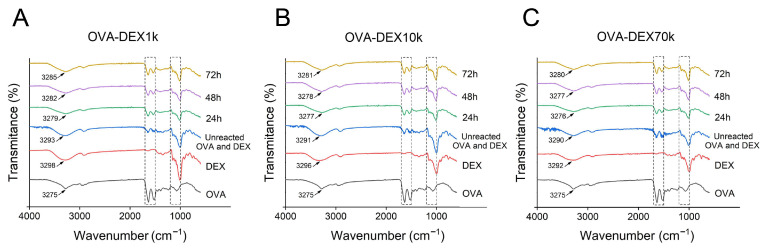
Fourier transform infrared spectra analysis. (**A**) FTIR spectra of native OVA, DEX1k, mixture of unreacted OVA and DEX1k, and OVA–DEX1k conjugates. (**B**) FTIR spectra of native OVA, DEX10k, mixture of unreacted OVA and DEX10k, and OVA–DEX10k conjugates. (**C**) FTIR spectra of native OVA, DEX70k, mixture of unreacted OVA and DEX70k, and OVA–DEX70k conjugates.

FT-IR spectroscopy is widely used to study the functional groups and chemical bonds in molecules, as well as the composition of the secondary structure of proteins [44]. In the range of 3300 to 3200 cm^−1^, free hydroxyl had stretching vibration absorption [17]. As shown in Figure 2, the conjugates exhibited a peak broadening and a band shift towards higher wavenumbers compared to OVA at wavenumbers of 3300–3200 cm^−1^. This could be attributed to the formation of covalent bonds between the protein and the polysaccharide, which increased the hydroxyl content in the conjugates [17]. The characteristics of the absorption band of the Amide I (1700–1600 cm^−1^) and Amide II (1600–1500 cm^−1^) were associated with the C=O stretching vibration, the C-N stretching, and the N-H bending of proteins, respectively [46,47]. The absorption band observed at the range of 1200–1000 cm^−1^ corresponded to the stretching vibration of the polarity C-O bond [17]. The characteristic absorption within the range of 1200–1000 cm^−1^ was enhanced with different degrees in the OVA–DEX conjugates compared to pure OVA, confirming the successful access of DEX molecules to the protein molecules through covalent bonding in the conjugates [17]. Fourier self-deconvolution and Gaussian function were then utilized to analyze the secondary structure of amide I band (Figure S1). The results showed that the secondary structure of the conjugates presented an increase in α-helices and random coil compared with OVA (Table S1), which was consistent with the previous results of Yan et al. [35].

#### 3.1.3. Surface Hydrophobicity

**Figure 3:**
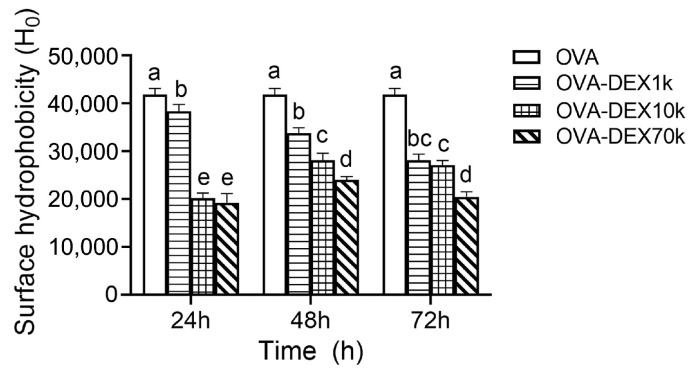
Surface hydrophobicity of OVA–DEX conjugates with different molecular weights after various reaction times. Different letters have significant differences (*p* < 0.05).

The surface hydrophobicity has an important impact on the surface properties of proteins. For OVA, over half of the 385 amino acid residues are hydrophobic, and these residues are hidden inside the protein. When the hydrophobic groups were exposed to a polar environment or the structure of protein unfolds, the surface hydrophobicity of the protein would change [48]. As shown in Figure 3, compared with OVA, the surface hydrophobicity of OVA–DEX with different molecular weights gradually decreased with the prolonged reaction time of the Maillard reaction. This could be attributed to the fact that the highly branched DEX brings in more hydrophilic hydroxyl groups, which further increased the hydrophilicity of the OVA and reduced the surface hydrophobicity. The results showed that, after 72 h of the Maillard reaction, the surface hydrophobicity of the conjugates was significantly reduced compared to OVA (*p* < 0.05), since DEX made it more difficult for the ANS probe to bind to the hydrophobic regions inside the OVA. In addition, as a result of heating, the OVA protein unfolded and the hydrophobic structure exposed, which further reduced the surface hydrophobicity of the OVA protein after reacting with DEX.

#### 3.1.4. Heat Stability and Thermal Analysis

**Figure 4:**
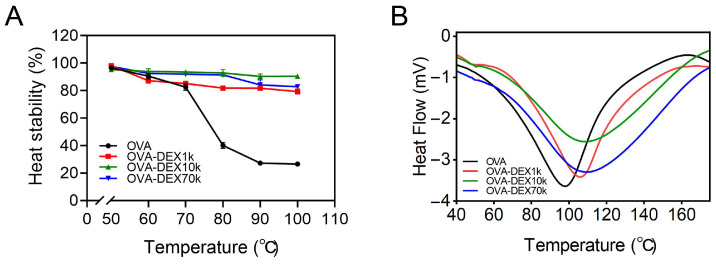
The (**A**) heat stability and (**B**) DSC diagram of OVA and OVA–DEX conjugates.

As previously reported, OVA might form aggregates and even a gel when subjected to high temperatures due to the heat-denaturation-coupled aggregation of OVA [34,49,50]. Thus, studying the heat stability of OVA and OVA–DEX conjugates is important in evaluating their ability to maintain the thermal stability of the emulsion. As shown in Figure 4A, when the temperature was below 70 °C, fewer proteins were precipitated from the system, indicating that OVA and OVA–DEX could maintain the stability of the system. However, as the temperature exceeded 70 °C, the stability of OVA decreased, which is in agreement with the results of a previous report [50]. The OVA–DEX conjugates were soluble after heating the solution at 100 °C for 10 min, whereas most of the intact OVA was precipitated at 90 °C. Consistently with previous research [34], the results revealed that the DEX modification of OVA might be effective in improving the heat stability of the protein. Furthermore, the DSC analysis was used to determine the thermal properties of OVA and OVA–DEX conjugates. As shown in Figure 4B, OVA exhibited a peak transition at 97.9 °C, which was in line with the previous research [17,51]. The peak transition temperature (Tp) of OVA–DEX conjugates was 105.9, 108.9, and 109.5 °C for the OVA–DEX1k, OVA–DEX10k, and OVA–DEX70k conjugates, respectively; this means that the thermal stability of OVA was improved by glycosylation modification. The previous research conducted by Geng et al. discovered similar findings, specifically indicating that conjugation with carboxymethyl cellulose increased the denaturation temperature of OVA [17]. Overall, the heat stability and thermal analysis revealed that covalent grafting of DEX could enhance the heat stability and increase the denaturation temperature of the OVA protein.

### 3.2. Fabrication and Characterization of Emulsion

#### 3.2.1. Encapsulation Efficiency

**Figure 5:**
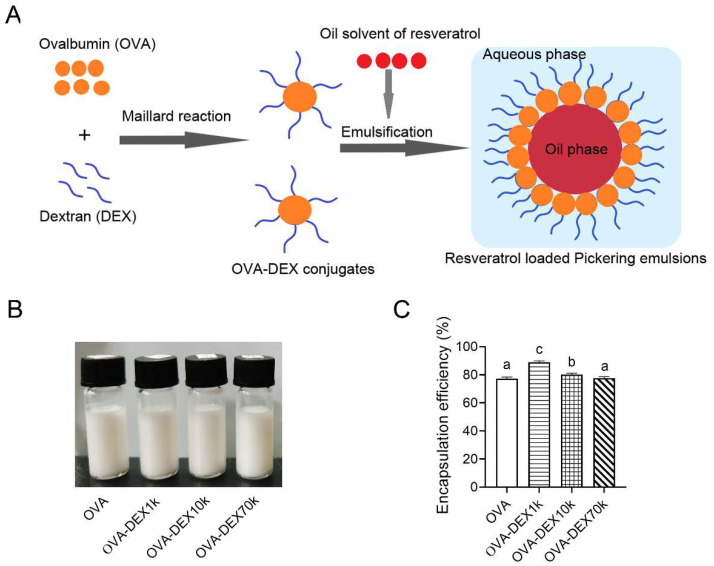
(**A**) Synthesis scheme of the OVA–DEX-stabilized emulsions. (**B**) The representative appearance photographs of emulsions prepared by OVA and OVA–DEX conjugates. (**C**) The encapsulation efficiency of resveratrol in the emulsions stabilized by OVA, OVA–DEX1k, OVA–DEX10k, and OVA–DEX70k conjugates. Different letters have significant differences (*p* < 0.05).

Encapsulation efficiency is a crucial parameter in determining the quality of emulsion. The emulsions embedded with resveratrol were prepared using high-pressure homogenization based on the scheme depicted in Figure 5A. The appearance photograph was presented in Figure 5B and the EEs of emulsions stabilized with OVA, OVA–DEX1k, OVA–DEX10k, and OVA–DEX70k conjugates were determined, as shown in Figure 5C. The results demonstrated that the encapsulation efficiency of resveratrol in the OVA-stabilized emulsion was only 77.3%, which was remarkably low compared with those of the emulsions prepared with the OVA–DEX1k or OVA–DEX10k conjugates. Particularly, the emulsion stabilized with OVA–DEX1k exhibited a much greater efficiency in encapsulating resveratrol, with an EE value of 89.0%. These findings indicated that OVA–DEX conjugates were beneficial for the encapsulation of resveratrol, and the conjugates with a higher degree of grafting had a superior encapsulation effect on resveratrol.

#### 3.2.2. Storage Stability

**Figure 6:**
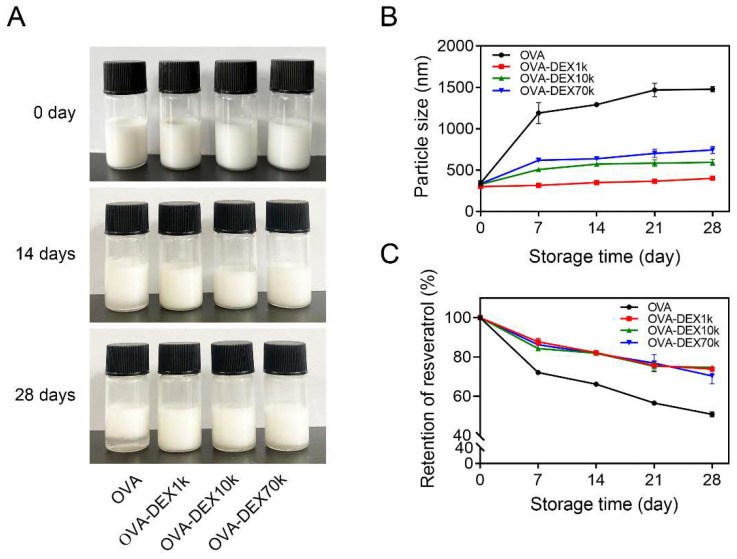
(**A**) The representative appearance photographs of emulsions stored at 25 °C for a specified period of time. (**B**) The average particle size of the emulsions stabilized by OVA and OVA–DEX conjugates during different storage time. (**C**) Retention of resveratrol in the emulsions stabilized by OVA and OVA–DEX conjugates after storage at 25 °C for the indicated time.

The freshly produced emulsions prepared by OVA, unreacted OVA and DEX, and the OVA–DEX conjugates were uniform in appearance and had particle sizes of 301.8–343.3 nm (Figure 6A,B and Figure S2). After storage at 25 °C for 14 days, the average particle size of the OVA-stabilized emulsion increased to 1291.7 nm and the emulsion exhibited a more pronounced phase separation, indicating a tendency for particle aggregation into larger sizes (Figure 6A,B). The emulsion prepared using unreacted OVA and DEX as emulsifier system displayed an average particle size of 846.3 nm (Figure S2A,B). As for the emulsions made by the OVA–DEX1k, OVA–DEX10k, and OVA–DEX70k conjugates, the droplet size increased to 350.3 nm, 573.3 nm, and 638.7 nm, respectively, which were significantly lower than that of the OVA-stabilized emulsion or the DEX-stabilized emulsion (Figure 6B). Consistently with previous research, the results demonstrated that the storage stability of emulsions was enhanced by addition of polysaccharides, and protein–polysaccharide-conjugate-stabilized emulsions displayed even better stability during storage at 25 °C [52]. When stored at 25 °C for a duration of 28 days, the OVA–DEX-stabilized emulsions displayed a much smaller droplet size compared to the OVA-stabilized emulsion. It is worth noting that the emulsion created using the OVA–DEX1k conjugate exhibited the smallest average particle size of 402.0 nm, even after being stored for 28 days. This improved storage stability could be attributed to the fact that the Maillard reaction that occurred between the polysaccharides and the proteins increased the repulsion effect between the particles in the emulsion, thereby forming a stable system that prevented the excessive coagulation of the particles [28]. Overall, these findings suggested that DEX effectively prevented particle aggregation in the emulsion, and the OVA–DEX1k conjugate performed better in terms of stability under room temperature.

Next, we examined the effect of the OVA–DEX conjugates on the retention of resveratrol during long-term storage at room temperature. As shown in Figure 6C and Figure S2C, after storage at 25 °C for 7 days, the retention of resveratrol in the emulsions prepared by OVA, unreacted OVA and DEX, OVA–DEX1k, OVA–DEX10k, and OVA–DEX70k was 72.1%, 76.0%, 87.7%, 84.3%, and 86.3%, respectively. The higher retention of resveratrol in the OVA–DEX-stabilized emulsion was probably due to the fact that the dense network formed by copolymer reduced the precipitation of oil solution that encapsulated resveratrol, thereby preventing the oxidative degradation of resveratrol [26]. After 28 days of storage at 25 °C, the emulsion stabilized solely with OVA showed faster decomposition of resveratrol, with only 50.8% retention of resveratrol remaining. In contrast, the use of OVA–DEX conjugates as the emulsifiers resulted in the retention of approximately 73% for resveratrol (Figure 6C). Considering the results of the average particle size of the emulsions, using OVA–DEX conjugates as emulsifiers enhanced the stability of the emulsion and reduced the degradation of resveratrol during storage at room temperatures, thereby ensuring better long-term stability.

#### 3.2.3. Physicochemical Stability

##### Effect of Thermal Treatment

**Figure 7:**
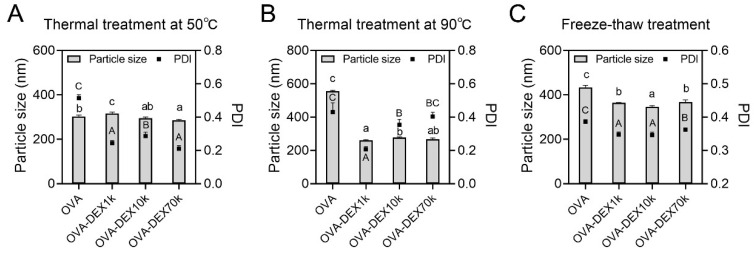
The particle size of the emulsions stabilized by OVA and OVA–DEX conjugates under different thermal treatments of (**A**) 50 °C for 30 min, (**B**) 90 °C for 30 min, and (**C**) freeze–thawing. Different letters have significant differences (*p* < 0.05).

The O/W emulsions are thermodynamically unstable systems and can easily be disrupted by environmental factors [53]. In this study, the effects of different thermal treatments at 50 °C, 90 °C, and freeze–thawing on the stability of emulsions were investigated. As shown in Figure 7A, the particle sizes between the emulsions had diminutive differences, whereas the PDI values of emulsions stabilized by the OVA–DEX conjugates were notably lower than that of the OVA-stabilized emulsion. After thermal treatment at 90 °C, the droplet size of the OVA-stabilized emulsion increased to 555.3 nm, while the particle size of the OVA–DEX prepared emulsions remained below 300 nm (Figure 7B). It is important to note that, even after the heating treatment at 90 °C, the PDI value of the OVA–DEX1k emulsion remained around 0.2, suggesting that the stability of the OVA–DEX1k emulsion was maintained (Figure 7B). Such a condition might be attributed to the following factors: the grafted DEX created a barrier layer around the proteins, preventing them from coming into direct contact and forming aggregates when exposed to thermal treatment [54]. Moreover, the conjugated DEX might form a thick interface layer as the protective coating on the surface of oil droplets, which weakened the sensitivity of the proteins to high temperatures [55].

The freeze–thaw treatment may cause high instability of the oil phase in the emulsion system, leading to the phase separation of the emulsion. The addition of polysaccharides in the emulsion might increase the adsorption of oil droplets, therefore reducing their precipitation [56]. The results of freezing and thawing treatment of the emulsion revealed that the particle size and PDI value of the OVA-stabilized emulsion were 433.7 nm and 0.39, respectively, which were markedly higher than those of the OVA–DEX-prepared emulsions (Figure 7C). This indicated that the inclusion of the OVA–DEX conjugates in the emulsion formulation enhanced the stability of the resveratrol emulsion under freeze–thaw conditions. The above results demonstrated that the emulsions prepared by the OVA–DEX conjugates exhibited better stability under various thermal conditions, including 50 °C and 90 °C heating treatments, as well as the freeze–thaw treatment.

##### Effect of pH

**Figure 8:**
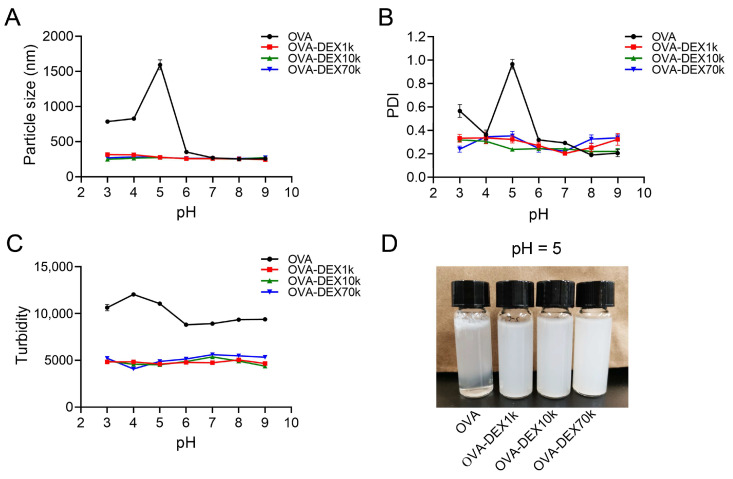
Effects of different pH values on (**A**) particle size, (**B**) PDI, and (**C**) turbidity of emulsions stabilized by OVA and OVA–DEX conjugates. (**D**) The representative image of emulsions at a pH value of 5.

The stability of the emulsions under different pH environmental treatments becomes crucial to for their application in food processing and in vivo digestive systems. As shown in Figure 8A,B, within the pH range of 3–5, the average particle size and PDI of the OVA-stabilized emulsion increased significantly. This suggested that OVA underwent deformation or even aggregation at low pH levels, leading to noticeable phase separation in the emulsion. Fortunately, the particle size of the emulsion prepared using OVA–DEX conjugates as an emulsifier remained between 200 and 300 nm, and the PDI value was also within the acceptable range of 0.2–0.4 (Figure 8A,B). In addition, considering the turbidity (T) value, it was observed that, within the pH range of 3–5, the T value of the OVA emulsion increased significantly, indicating a decrease in the overall emulsion stability (Figure 8C). The isoelectric point of OVA was reported to be approximately 4.5–4.7; here, OVA was electrically neutral and might lose its emulsifying capability, leading to droplets aggregation or creaming [16,57,58]. As depicted in Figure 8D and Figure S3, when the pH value was around the isoelectric point of OVA, the emulsion prepared with OVA and the unreacted OVA–DEX mixture showed evident phase separation, while the emulsions prepared with the OVA–DEX copolymers remained homogeneous and stable. The average particle size and PDI of the emulsion prepared by the unreacted OVA and DEX were markedly decreased compared with OVA-stabilized emulsion. As expected, the OVA–DEX conjugate-stabilized emulsions exhibited even lower particle size and PDI. This could be due to the fact that the addition of DEX to OVA-stabilized emulsions, either conjugated or unconjugated with the proteins, improved the pH stability of emulsions [59,60,61]. Consistently with previous reports, the above results indicated that the emulsions stabilized using the protein and polysaccharide complexes prepared by Maillard reaction showed high tolerance when the pH was close to the isoelectric point of 4.5–4.7 [26,52]. Within the pH range of 6–9, although the particle size and PDI of the emulsion tended to stabilize, the T values of the OVA–DEX copolymer emulsions were notably lower than that of the OVA-stabilized emulsion, indicating that the stability of the OVA–DEX-stabilized emulsions was superior to that of the OVA-stabilized emulsion. One possible explanation for this improvement was that the hydrophilic polysaccharides present on the surface of the emulsion delivery system played a crucial role in providing strong steric repulsion, thus preventing droplet aggregation [62]. Therefore, using OVA–DEX conjugates as emulsifiers could enhance the stability of emulsions specifically at low pH and improve their resistance to the destabilizing effects caused by changes in pH value.

##### Effect of Ionic Strength

**Figure 9:**
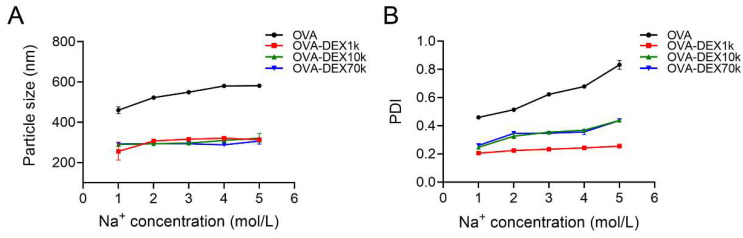
Effects of different ionic strength on (**A**) particle size and (**B**) PDI of emulsions stabilized by OVA and OVA–DEX conjugates.

Na^+^ is commonly used as a food additive in the food processing and handling industry. Therefore, it is important to understand the effects of different Na^+^ concentrations on the stability of emulsions. Figure 9 showed that as the Na^+^ concentration increased, the average particle size and PDI values of the resveratrol emulsion increased gradually. Among the different emulsions, the emulsion embedded with OVA exhibited higher droplet size and PDI than the OVA–DEX-stabilized emulsion, indicating the weaker resistance of OVA-stabilized emulsion to Na^+^. Specifically, when exposed to 5 mol/L Na^+^, the particle size of the emulsion followed the following order: OVA > OVA–DEX70k > OVA–DEX10k > OVA–DEX1k. The PDI value of the OVA-stabilized emulsion reached 0.83. However, the PDI values of the OVA–DEX70k and OVA–DEX10k-stabilized emulsions fell between 0.25 and 0.50, and the PDI of OVA–DEX1k-stabilized emulsion remained within the narrower range of 0.10 to 0.25. It was hypothesized that the higher degree of grafting DEX contributed to increased ion resistance in the emulsion, which enhanced the stability of the emulsion in high-Na^+^-concentration environments. In combination with the results of the droplet size and PDI, it could be seen that OVA–DEX conjugates, especially the OVA–DEX1k conjugate, maintain the better stability of the emulsion in high Na^+^ concentrations.

#### 3.2.4. Antioxidant Activity Test

**Figure 10:**
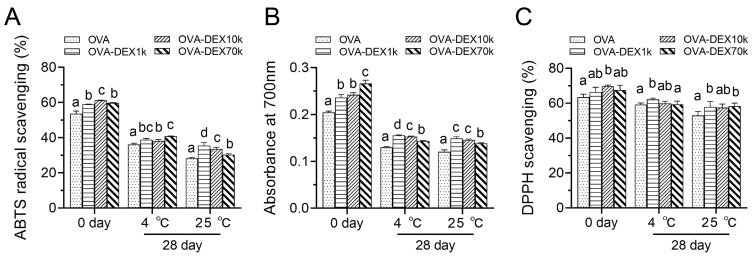
(**A**) ABTS radical scavenging activity, (**B**) reducing power, and (**C**) DPPH scavenging capacity of emulsions stabilized by OVA and OVA–DEX conjugates under different storage conditions. Different letters have significant differences (*p* < 0.05).

The antioxidant properties of different samples were evaluated by the methods of reducing power test, ABTS radical scavenging, and DPPH scavenging, and the results are illustrated in Figure 10. For the newly produced emulsions, the OVA–DEX-stabilized resveratrol emulsions exhibited higher ABTS radical scavenging and stronger reducing power compared with the OVA-stabilized emulsion. The emulsion prepared using OVA–DEX10k also showed remarkably improved DPPH scavenging ability compared with the OVA-stabilized emulsion. After a long period of storage in the dark, the antioxidant activity of the emulsions diminished to varying degrees. Nonetheless, there was a noticeable trend in which the OVA–DEX-stabilized emulsions exhibited enhanced antioxidant properties compared to the OVA-stabilized emulsions when subjected to identical storage conditions. Specifically, when the emulsion was prepared using OVA–DEX1k, the highest antioxidant capacity was achieved for all the three methods after 28 days of storage at 25 °C. This phenomenon could be attributed to several factors. Firstly, the OVA–DEX-stabilized emulsion exhibited a superior encapsulation efficiency of resveratrol, leading to a higher resveratrol content in the emulsion, thereby enhancing its antioxidant ability [63,64]. In addition, the covalent grafting of DEX onto OVA protein enhanced the repulsion between droplets in the emulsion, preventing droplet aggregation and improving the stability of the emulsion [28]. As a result, it prevented resveratrol leakage in the oil solution during storage and thus inhibited the oxidation degradation of resveratrol [65]. Moreover, the amount of Maillard reaction products was increased with the increase in browning of the samples, which resulted in higher antioxidant activity [66]. Therefore, it could be concluded that OVA–DEX-stabilized emulsions displayed enhanced ABTS cation radical, stronger reducing activity, and DPPH scavenging ability, indicating that OVA–DEX conjugates contributed to maintaining the antioxidant properties of resveratrol-loaded emulsion.

#### 3.2.5. Rheological Properties Analysis

**Figure 11:**
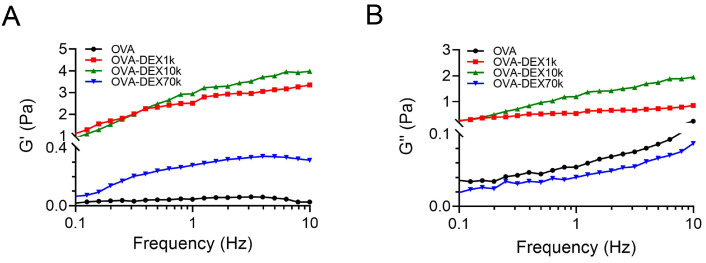
(**A**) Storage modulus and (**B**) loss modulus on frequency for emulsions stabilized by OVA and OVA–DEX conjugates.

The oscillation test was conducted to gain a better understanding of the relationship between the internal structure and flow characteristics, such as viscosity and rheological behavior. The results showed that, as the oscillatory frequency increased, both the G’ and G” values of the emulsions gradually increased, implying that the particles in the emulsions became more dispersed and the viscosity of the emulsions decreased [67]. The results of the shear scanning analysis revealed that the emulsions stabilized with OVA–DEX1k and OVA–DEX10k exhibited higher values of storage modulus and loss modulus compared to the emulsion prepared with OVA (Figure 11 and Figure S4). This phenomenon indicates that the OVA–DEX1k- and OVA–DEX10k-stabilized emulsions had increased viscosity and were able to slow down the rise of droplets, thereby enhancing the overall stability of the emulsion. Moreover, the OVA–DEX conjugates, especially OVA–DEX1k and OVA–DEX10k, have significant potential in improving the stability of the emulsions.

## 4. Conclusions

In this study, OVA–DEX conjugates were prepared through the Maillard reaction; then, SDS-PAGE analysis, FTIR, and surface hydrophobicity and DSC analyses were performed to assess the structure and characteristics of the conjugates. Subsequently, resveratrol-loaded emulsions were produced using the high-pressure homogenization method, with OVA or OVA–DEX conjugates serving as emulsifiers. The encapsulation of resveratrol emulsions stabilized by OVA–DEX conjugates was higher than that of the emulsions stabilized by OVA; the physical stability of the emulsions during long-term storage at 25 °C was enhanced by the OVA–DEX conjugates. This study also demonstrated that the capabilities of OVA–DEX-conjugate-stabilized emulsions against physicochemical treatments, such as thermal treatment, changes of pH 3–9, and 1–5 mol/L NaCl solutions, were markedly improved compared with emulsions stabilized by OVA alone. Furthermore, the reducing power test and ABTS radical scavenging assay indicated that the antioxidant capacity of resveratrol-loaded emulsions prepared by OVA–DEX conjugates was sensibly enhanced in the newly produced emulsions as well as the emulsions stored after 28 days, indicating that OVA–DEX conjugates contributed to maintaining the antioxidant properties of resveratrol. The results of this study proved that OVA–DEX conjugates were excellent emulsifiers due to their notable thermal stability and significant effect in enhancing the physicochemical stability and antioxidant activity of the resveratrol-loaded emulsions.

## Data Availability

The original contributions presented in the study are included in the article/Supplementary Material, further inquiries can be directed to the corresponding author.

## Supplementary Materials

The following supporting information can be downloaded at: https://www.mdpi.com/article/10.3390/foods13081246/s1, Table S1: Secondary structure of native OVA and OVA–DEX conjugates with different molecular weights after various reaction time. Figure S1: The deconvolution of the amide-I band of OVA. Figure S2. Storage stability of OVA-, unreacted OVA and DEX10k-, and OVA–DEX10k-conjugate-stabilized emulsions. Figure S3. Particle size, PDI, and representative image of emulsions stabilized by OVA, unreacted OVA and DEX10k, and OVA–DEX10k conjugates at a pH value of 5. Figure S4: The strain sweep test of OVA-stabilized emulsion over a strain range of 0.01–100% at a fixed frequency of 1 Hz.
