# Supplementary material for: Enhancing Stability and Antioxidant Activity of Resveratrol-Loaded Emulsions by Ovalbumin–Dextran Conjugates
Source: Foods. 2024 Apr 19;13(8):1246. doi: 10.3390/foods13081246 (PMC11049361; doi:10.3390/foods13081246)
Supplement: Supplementary file 1 [file foods-13-01246-s001.zip › Supplementary Figure.docx]

**Supplementary Figures**


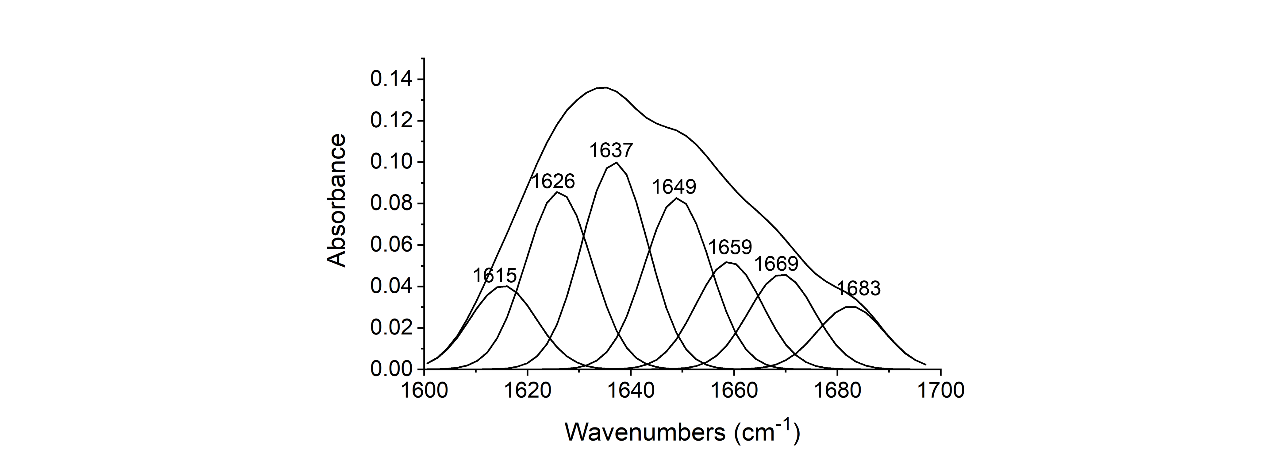


Figure S1. The deconvolution of the amide-I band of OVA.


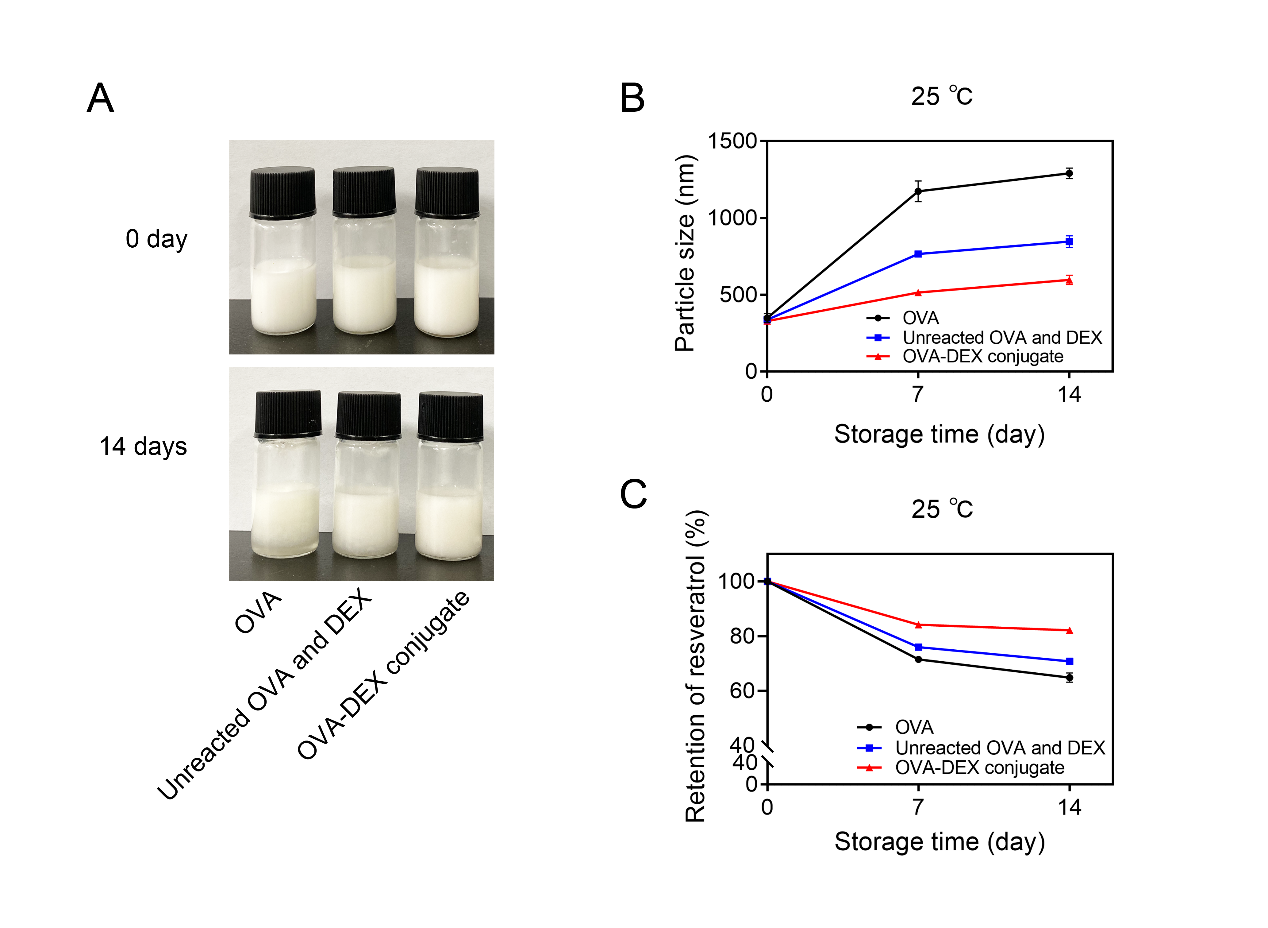


Figure S2. Storage stability of OVA-, unreacted OVA and DEX10k-, and OVA-DEX10k conjugates-stabilized emulsions. (A) The representative appearance photographs of emulsions stored at 25 ℃ for 14days. (B) The average particle size of the emulsions stabilized by OVA, unreacted OVA and DEX10k, and OVA-DEX10k conjugates during different storage time. (C) Retention of resveratrol in the emulsions stabilized by OVA, unreacted OVA and DEX10k, and OVA-DEX10k conjugates after storage at 25 ℃ for the indicate time.


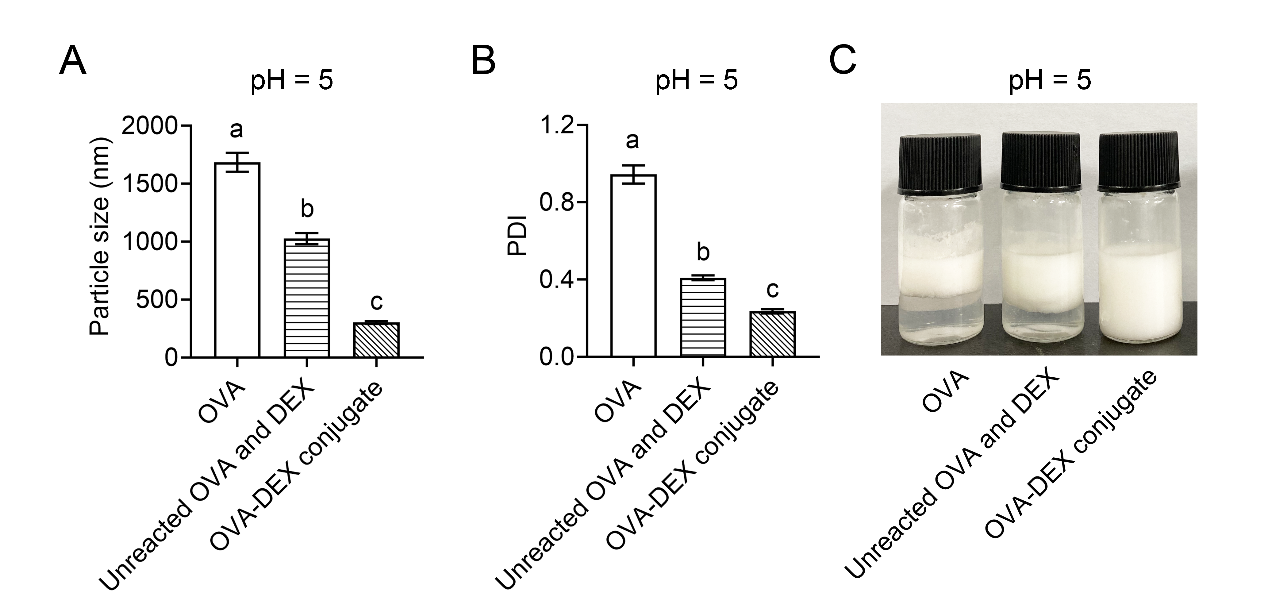


Figure S3. (A) Particle size, (B) PDI, and (C) representative image of emulsions stabilized by OVA, unreacted OVA and DEX10k, and OVA-DEX10k conjugates at a pH value of 5.


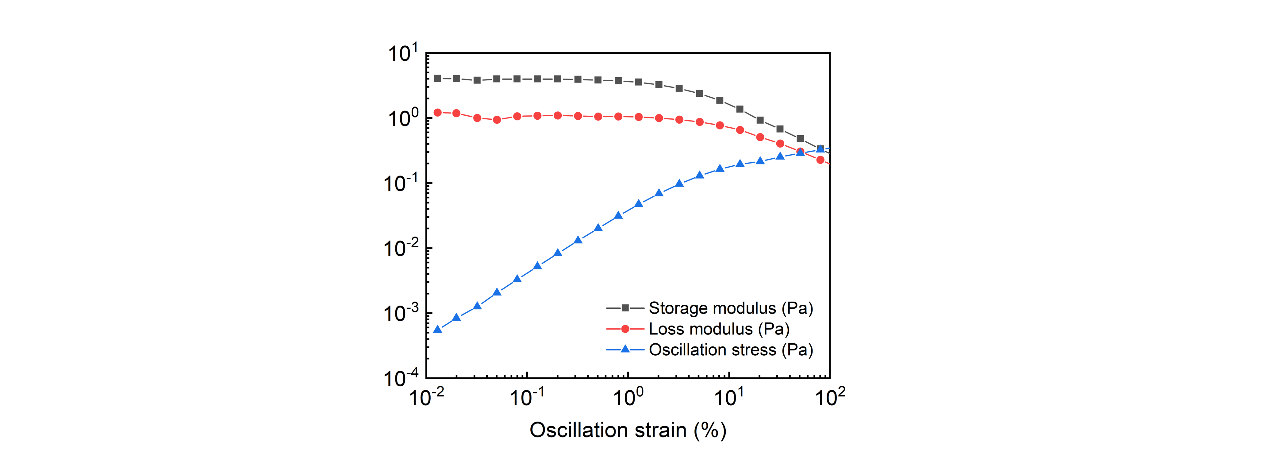


Figure S4. The strain sweep test of OVA-stabilized emulsion over a strain range of 0.01-100 % at a fixed frequency of 1 Hz.
