# Supplementary material for: Enhancing Stability and Antioxidant Activity of Resveratrol-Loaded Emulsions by Ovalbumin–Dextran Conjugates
Source: Foods. 2024 Apr 19;13(8):1246. doi: 10.3390/foods13081246 (PMC11049361; doi:10.3390/foods13081246)
Supplement: Supplementary file 1 [file foods-13-01246-s001.zip › Supplementary Tables.docx]

**Supplementary Tables**

**Table S1.** Secondary structure of native OVA and OVA-DEX conjugates with different molecular weights after various reaction time.

| Reaction time | Sample | α-helix (%) | β-sheet (%) | β-turn (%) | Random coil (%) |
| --- | --- | --- | --- | --- | --- |
|  | native OVA | 10.31±0.65 | 33.56±1.32 | 45.71±2.08 | 10.42±0.32 |
| 24 h | OVA-DEX1K | 11.69±0.75^ab^ | 30.45±1.90^ab^ | 47.33±2.91^b^ | 10.53±0.29^a^ |
|  | OVA-DEX10K | 11.77±0.11^ab^ | 27.30±0.38^a^ | 47.24±0.65^b^ | 13.69±0.18^ab^ |
|  | OVA-DEX70K | 11.30±0.79^a^ | 37.88±3.45^c^ | 40.45±1.03^a^ | 10.37±1.76^a^ |
| 48 h | OVA-DEX1K | 10.77±0.7^a^ | 32.52±5.02^c^ | 43.54±5.19^a^ | 13.17±0.44^b^ |
|  | OVA-DEX10K | 12.30±0.16^a^ | 33.07±0.27^abc^ | 43.11±0.63^b^ | 11.52±0.23^a^ |
|  | OVA-DEX70K | 11.33±1.6^a^ | 34.07±1.6^bc^ | 42.07±0.97^ab^ | 12.54±0.73^b^ |
| 72 h | OVA-DEX1K | 12.78±0.23^c^ | 29.33±0.68^a^ | 45.98±0.78^cd^ | 11.91±0.35^b^ |
|  | OVA-DEX10K | 12.06±0.27^bA^ | 33.44±0.68^bc^ | 43.17±0.60^ab^ | 11.33±0.19^b^ |
|  | OVA-DEX70K | 12.62±0.21^bc^ | 31.34±0.64^ab^ | 44.48±0.53^bc^ | 11.55±0.13^b^ |

Different letters have significant differences (*P* < 0.05).
